# Graph analysis of dream reports is especially informative about psychosis

**DOI:** 10.1038/srep03691

**Published:** 2014-01-15

**Authors:** Natália B. Mota, Raimundo Furtado, Pedro P. C. Maia, Mauro Copelli, Sidarta Ribeiro

**Affiliations:** 1 Brain Institute, Federal University of Rio Grande do Norte (UFRN), Natal, Brazil, Postal Code: 59056-450; 2 Physics Department, Federal University of Pernambuco (UFPE), Recife, Brazil, Postal Code: 50670-901; 3 Shared corresponding authorship.

## Abstract

Early psychiatry investigated dreams to understand psychopathologies. Contemporary psychiatry, which neglects dreams, has been criticized for lack of objectivity. In search of quantitative insight into the structure of psychotic speech, we investigated speech graph attributes (SGA) in patients with schizophrenia, bipolar disorder type I, and non-psychotic controls as they reported waking and dream contents. Schizophrenic subjects spoke with reduced connectivity, in tight correlation with negative and cognitive symptoms measured by standard psychometric scales. Bipolar and control subjects were undistinguishable by waking reports, but in dream reports bipolar subjects showed significantly less connectivity. Dream-related SGA outperformed psychometric scores or waking-related data for group sorting. Altogether, the results indicate that online and offline processing, the two most fundamental modes of brain operation, produce nearly opposite effects on recollections: While dreaming exposes differences in the mnemonic records across individuals, waking dampens distinctions. The results also demonstrate the feasibility of the differential diagnosis of psychosis based on the analysis of dream graphs, pointing to a fast, low-cost and language-invariant tool for psychiatric diagnosis and the objective search for biomarkers. The Freudian notion that “dreams are the royal road to the unconscious” is clinically useful, after all.

Differential diagnosis in psychiatry is more often than not a difficult task, unsupported by objective tests and necessarily performed by experts^1^. Standard psychiatric diagnosis has been harshly criticized, despite century-old efforts towards an accurate classification of mental illnesses^1^,^2^,^3^,^4^. Multi-site and cross-cultural expert agreement is low, most diseases do not have unequivocal biomarkers, and clear-cut distinctions between certain maladies may be unwarranted^5^,^6^. For instance, subjects with schizophrenia or bipolar disorder type I may share several positive psychotic symptoms such as hallucinations, delusions, hyperactivity and aggressive behavior^7^.

The development of quantitative methods for the evaluation of psychiatric symptoms offers hope to overcome this foggy scenario^8^,^9^. In particular, we have recently shown that the graph-theoretical analysis of dream reports produced by psychotic patients can separate schizophrenic from manic subjects^10^. This was possible because their speech features are usually quite different. Schizophrenic subjects frequently display negative symptoms including alogia, i.e. they speak laconically and with little digression^7^,^10^. Subjects with bipolar disorder, especially during the manic stage, tend to present the opposite symptom called logorrhea, with much recursiveness in association with positive symptoms^7^,^10^. These differences in symptomatology led us to hypothesize that schizophrenic and bipolar subjects would produce less connected word graphs than control subjects, in correlation with negative symptoms. It also remains unsettled whether dream reports are crucial for the differential diagnosis of psychosis, as early psychiatrists would have sustained^11^,^12^, or whether waking contents are equally informative.

To elucidate these issues, we quantified the speech graph attributes (SGA; Figure 1a, Figure 2) of dream and waking reports obtained from clinical oral interviews of schizophrenic, bipolar type I, and control subjects (Supplementary Table S1). Using a Bayesian classifier, we compared the differential diagnosis of psychosis provided by dream-related SGA, waking-related SGA or standard psychometric scores. Translation of the reports into five major Western languages was performed to assess language-related variations.

## Results

**Figure 1:**
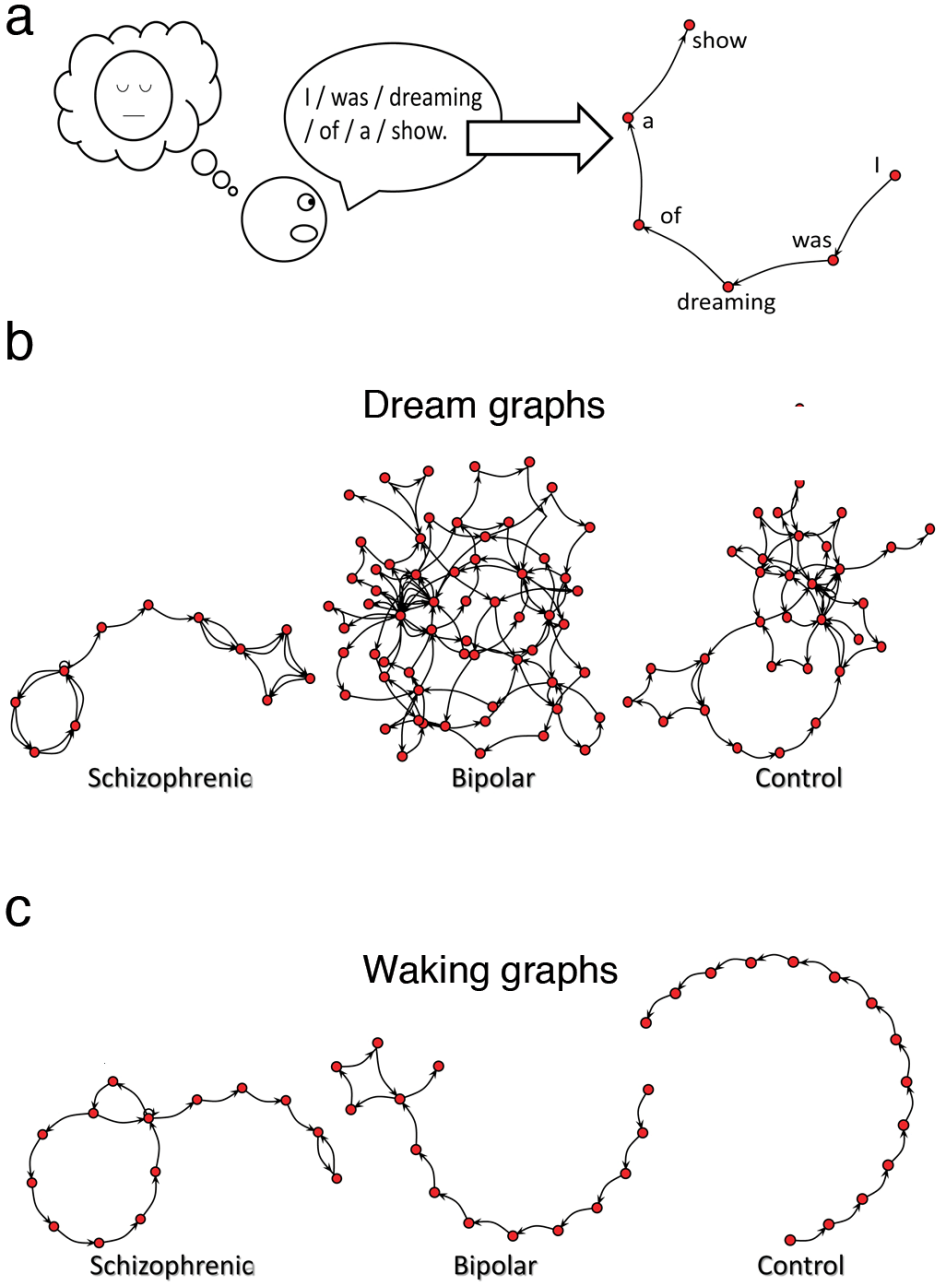
The speech graphs of schizophrenic, bipolar and control subjects are more varied for dream than for waking reports. (a) Graphs were generated from transcribed verbal reports using custom-made Java software (http://neuro.ufrn.br/softwares/speechgraphs). Drawing by NM. (b) Representative speech graphs extracted from dream reports from a schizophrenic, a bipolar and a control subject. (C) Same as in (b), but for waking reports of the same subjects.

Speech samples were recorded during psychiatric interviews as answers to two different requests: “Please report a recent dream” and “Please report your waking activities immediately before that dream”. Each report was transcribed and represented as a speech graph, in which every word represented a node, and every temporal connection between consecutive words represented an edge. The visual inspection of speech graphs suggests that dream reports (Figure 1b) vary more across groups than waking reports from the same subjects (Figure 1c).

A semantic and grammatical inspection of the most-frequent words, loops and their corresponding exit nodes showed few differences across dream and waking reports produced by psychotic and control subjects, with major overlap in word repertoire across groups (Supplementary Fig. S1). At the structural level, however, irrespective of meaning, clear contrasts emerged. While waking reports in all groups were typically sequential, with little recursiveness that reflected the linearity of chronological narrative, dream reports were quite convoluted when produced by bipolar and control subjects.

The SGA obtained for all the words in each report (Supplementary Tables S2 and S3) mostly agreed with the SGA obtained with smaller samples (n = 8 per group) and with the use of lexemes^10^, which require syntactical analysis. While dream-related graphs showed overall good classification quality and significant SGA differences between schizophrenic subjects and the two other groups (bipolar and control subjects), waking-related graphs failed to differentiate between any of the groups for any SGA (Figure 3a, Supplementary Table S4). We also found that nearly all SGA differed between dream and wake reports from bipolar and control subjects (Figure 3a).

Since schizophrenic subjects produce dream reports with a significantly smaller word count (WC) than dream reports produced by bipolar and control subjects, and given the fact that most SGA are strongly correlated with WC (Figure 4), it is possible that the differences between schizophrenic subjects and the two other groups derive solely from verbosity differences that could hinder the clinical applicability of the method. Indeed, bipolar and control subjects used more words than schizophrenic subjects when reporting a dream, making more complex graphs than when reporting on waking (Figure 3a). In contrast, schizophrenic subjects showed impoverished graphs for both dream and waking without any SGA difference between those, with overall low values of most SGA (Figure 3a).

To rule out the influence of verbosity, we analyzed the reports using a moving window of fixed word length (10, 20 and 30 words) with a step of 1 word. Each report yielded a population of graphs from which we calculated mean SGA. This procedure revealed that schizophrenic subjects yielded significantly less connected graphs (smaller LCC and LSC) and fewer edges (E) than bipolar and control subjects, for every word length tested and for both dream and waking (Figure 5a for word length = 30). Small graphs (word length = 10 and 20) showed smaller internal distances (Diameter and ASP) in schizophrenic subjects than in control subjects, for both dream (word length 10: Diameter P = 0.0001, ASP P = 0.0001; word length 20: Diameter P = 0.0007, ASP P = 0.0004) and waking (word length 10: Diameter P = 0.0021, ASP P = 0.0019; word length 20: Diameter P = 0.0013, ASP P = 0.0006). Additionally, dream-related small graphs had smaller ATD (word length 10 P = 0.0028; word length 20 P = 0.0106), and waking-related small graphs had smaller distances (word length 10 ASP P = 0.0140; word length 20 Diameter P = 0.0054, ASP P = 0.0043) in schizophrenic subjects, in comparison with bipolar subjects. Altogether the data show that reports from schizophrenic subjects, irrespective of originating from dream or waking, were characterized by small and poorly connected graphs, in comparison with bipolar and control subjects (Supplementary Table S2).

The reports produced by bipolar subjects, on the other hand, were very different depending on their source: dream events were reported with more recurrence (L3), and connectivity (ATD), higher density, smaller distances (diameter and ASP) and higher clustering coefficient (CC) than waking events (Figure 5a). Control subjects also reported dreams differently (with more E and larger LSC), and only schizophrenic subjects did not show any difference on dream or waking SGA (Figure 5a). When related to dreams, bipolar reports yielded less connected graphs (smaller LCC and LSC) with fewer nodes (N) than control subjects (Figure 5a). We also found graphs with smaller distances when using word length = 10 (Diameter P = 0.006, and ASP P = 0.0071), denoting smaller and less complex graphs in bipolar than in control subjects. None of these differences between bipolar and control subjects occurred in waking-related reports (Figure 5a).

To further explore dream versus waking differences in the reports of psychotic patients, we trained a Naïve Bayes classifier to differentiate among the groups using all SGA as inputs, with SCID results as golden standard. Schizophrenic subjects could be sorted from bipolar and control subjects with AUC between 0.6 and 0.86 for both dream and waking graphs (Figure 3b, Figure 5b, Supplementary Table S5), but only dream-related graphs could sort bipolar from control subjects (Figure 5b). Using raw data, it was possible to sort dream from waking reports among bipolar (AUC = 0.753) and control subjects (AUC = 0.807) (Figure 3c). Using an analysis window with length of 30 words, which provided the best accuracy for group classification, it was possible to automatically sort dream and waking reports among bipolar (AUC = 0.794) and control subjects (AUC = 0.65) (Figure 5c). This contrasts with reports from schizophrenic subjects, which showed no structural differences between dream and waking (Figure 3c, Figure 5c). Overall, the triple sorting of schizophrenic, bipolar and control subjects based on automatically selected attributes (E, LSC and ASP for dream reports; E and LCC for waking reports; word length = 30) was substantially better for dream-related SGA than for waking-related SGA or psychometric scores (Figure 5d).

The investigation of correlations between dream-related SGA and psychopathological symptoms grasped by PANSS and BPRS considering all 60 subjects produced interesting results: Using the attributes that best differentiated schizophrenic subjects from other groups (E, LCC and LSC), we found significant anti-correlations with negative and cognitive symptoms (Figure 6, Supplementary Fig. S2), known to be more frequent among schizophrenic subjects than among individuals with other psychotic syndromes^7^. Subjects that reported dream graphs with fewer edges or smaller connected components (LCC, LSC) scored higher on PANSS, on the negative PANSS subscale, and on PANSS questions regarding flattened affection, poor contact, difficulties on abstract thought, less spontaneous or fluent speech; these subjects also scored higher on BPRS questions about emotional retraction and flattened affection (Figure 6a). Significant anti-correlations in waking reports only occurred between LCC and general psychotic symptoms: Subjects that reported on waking with lower LCC presented higher scores on the PANSS question about judgment and critical capacity, and on the BPRS question regarding incoherent speech (Figure 6b).

Finally, to simulate the comparison of an actual psychiatric clinical assessment with a scenario in which graph analysis was employed, we compared the performances of binary classifiers trained with 1) selected SGA from both dreaming and waking, 2) PANSS and BPRS total scores, and 3) a combination of both. The attributes selected were those with significant correlation with psychometric scores: E, LCC and LSC for dream reports, and LCC for waking reports (Figure 6). We found that SGA sufficed to successfully sort the three groups, differentiating schizophrenic from control subjects with AUC = 0.941, bipolar from control subjects with AUC = 0.722, and schizophrenic subjects from bipolar subjects with AUC = 0.768 (Figure 7a). The psychometric scales were able to properly sort schizophrenic from control subjects (AUC = 0.955), and bipolar from control subjects (AUC = 0.935), but failed to differentiate schizophrenic subjects from bipolar subjects (AUC = 0.376). For a combination of SGA and standard scale scores, schizophrenic subjects were sorted from bipolar subjects with AUC = 0.748, bipolar subjects were sorted from control subjects with AUC = 0.928, and schizophrenic subjects were nearly perfectly sorted from control subjects with AUC = 0.993. Triple group sorting was better for SGA (AUC = 0.767) than for scales (AUC = 0.731), and was optimized by their combination (AUC = 0.849; Figure 7a). To assess the general applicability of the method, reports in Portuguese were translated to English, German, French, and Spanish. Figure 7b shows that group classification is remarkably similar across the five most prevalent Western languages.

## Discussion

The results provide a quantitative behavioral assessment of negative and cognitive symptoms, and thus demonstrate the feasibility of the automatic differential diagnosis of psychosis based on the word-by-word graph analysis of dream and waking reports. Rather than detracting from the classical distinction between schizophrenic and bipolar subjects, SGA quantitatively characterize their differences, providing a parameter space for the sorting of psychotic symptoms like alogia, logorrhea, lack of fluency on speech, and formal thought disorders (Figure 6). Thus, SGA analysis has potential to become a fast, non-invasive, low-cost and language-invariant tool for psychiatric diagnosis, by which a set of behavioral biomarkers could drive a more objective, bottom-up search for anatomical and physiological biomarkers^13^,^14^,^15^. Future research must follow up the investigation of non-medicated patients after first psychotic episodes, using longitudinal measures on same samples for prodrome and treatment evaluation^2^,^16^,^17^.

The results also show that dream reports are substantially more informative about the mental state of psychotic subjects than waking reports. The explanation for this fact, which echoes the centenary claim that dreams constitute a privileged window into thought^11^, may be rooted in the very introspective nature of dreams. While the episodic replay of recent waking activities occupies only 1–2% of dream reports^18^, declarative memories become more accessible for retrieval after REM sleep^19^, when most dreaming occurs^20^. Perhaps dream reports are more likely to reveal psychopathologies than waking reports because dreams are not proximally anchored on events shared with non-psychotic individuals, but rather on memories matured and restructured over time by the patient's own thought process.

Another important consideration is that dream events are more forgettable than waking events, probably because noradrenergic transmission is decreased during sleep^21^. On the other hand, REM sleep and dreaming are involved with emotional processing^22^,^23^. The combination of memory deficits with heightened emotional salience makes a request for a dream report yield more internally generated content than a request for a waking report. Importantly, patients with schizophrenia and bipolar disorder respond in opposite ways to the dream-report task: the former maintain their flattened speech, the latter confabulate even more.

Finally, it is possible that psychotic subjects are more likely to reveal the structure of their thinking when reporting on dreams simply due to the similarity between dreaming and psychosis^11^,^12^,^24^,^25^,^26^,^27^,^28^. The dream content in patients with schizophrenia is particularly affected by negative symptoms^29^, and their waking cognition matches the bizarreness of dream reports^27^, supporting dreaming as an experimental model of psychosis. SGA analysis combined with neural signal decoding during sleep^30^ and waking^31^ may soon allow for direct testing of these hypotheses.

## Methods

### Subjects

60 individuals (39 males and 21 females) independently diagnosed by the standard DSM IV ratings SCID^32^, as schizophrenic, bipolar type I, and control subjects (Supplementary Table S1). Study approved by the UFRN Research Ethics Committee (permit #102/06-98244); informed consent was obtained from all subjects.

### Clinical significance of the sample

Sample size was established according to the global and national prevalence of schizophrenia and bipolar disorder type I. Estimation of adequate sample size (N) considered the prevalence of Schizophrenia and Bipolar Disorder Type I according to the equation: 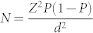
where Z = Z statistic for a level of confidence, P = expected prevalence or proportion and d = precision^33^. We adopted a conventional level of confidence of 95%, with Z = 1.96 (considering 95% of confidence interval) and a precision of d = 0.05^33^. A review of data from 46 countries with 154,140 cases considered the lifetime prevalence of schizophrenia to be 0.55% (±0.45 SD)^34^. The lifetime prevalence of bipolar disorder type I was considered to be 0.6% on a review of 61,392 cases from 11 countries^35^, or 0.9% (±0.2 SEM) based on an exclusive Brazilian sample on the same study^35^. The estimated sample sizes for the prevalences considered ranged from N = 1.53 to 15.21 for schizophrenia, and from N = 9.16 to 16.72 for bipolar disorder type I. Note that no estimated sample size was greater than N = 20, with N < 10 for mean lifetime prevalences in the world sample (schizophrenia 0.55% and bipolar type I 0.6%). Studies focused on the Brazilian population report a local prevalence of 0.57% for schizophrenia^36^, and a range of 0.3%–1.1% for bipolar disorder^37^. To ensure the clinical relevance of the results with equal size samples for each group (schizophrenia, bipolar and control), we selected N = 20 per group.

### Graph analysis of dream and waking reports

**Figure 2:**
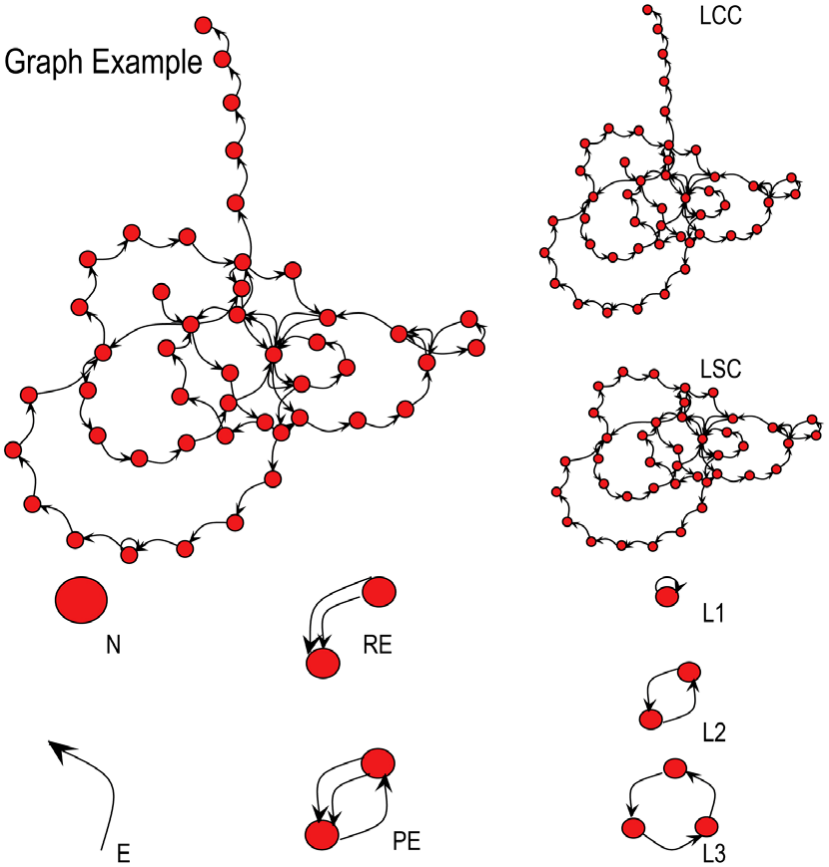
Speech Graph Attributes (SGA). Examples of speech graph attributes described in Methods.

**Figure 3:**
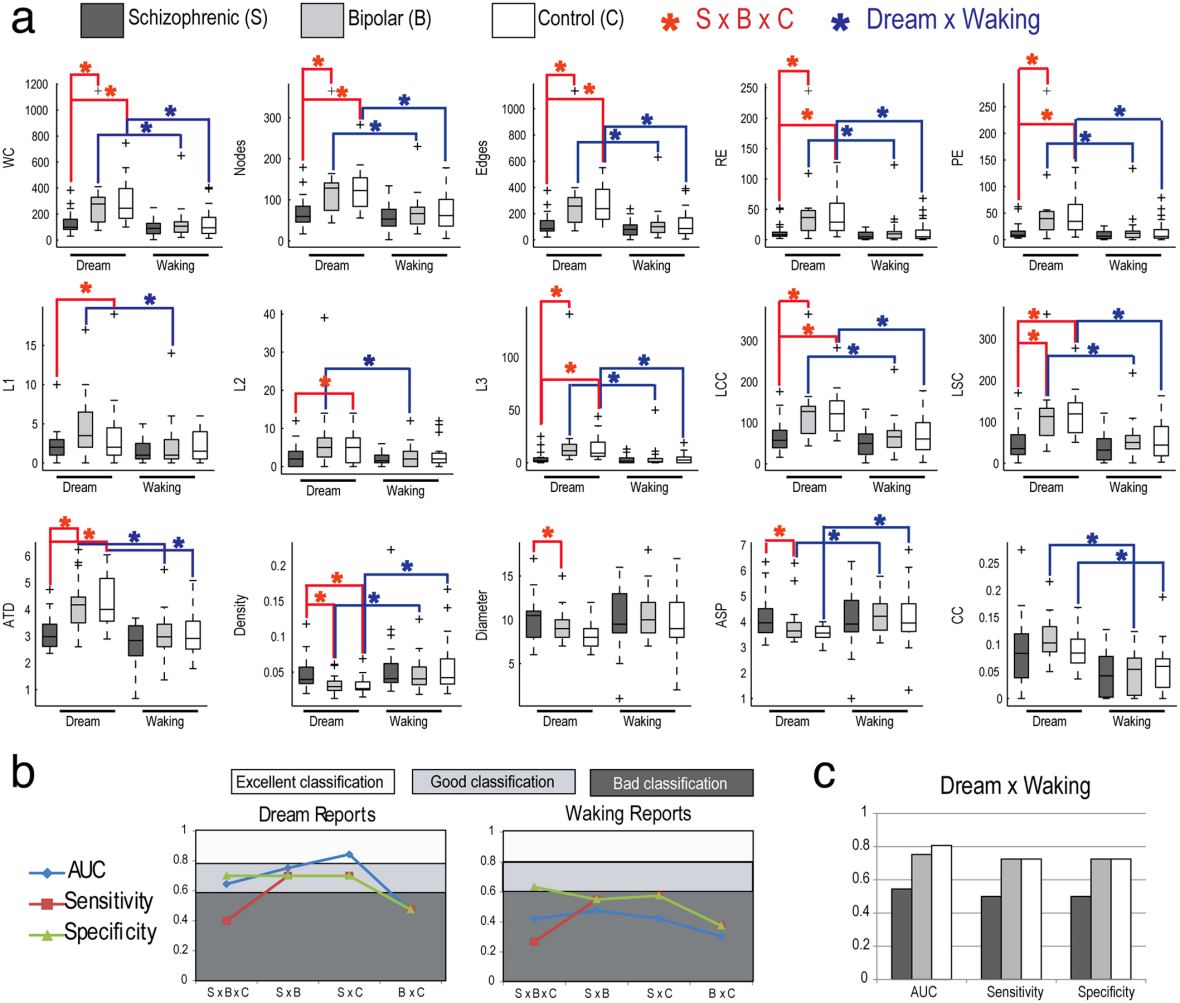
SGA using raw data (full reports) differentiate psychopathological groups. (a) SGA boxplots with significant differences among schizophrenic, bipolar and control groups indicated in red, and significant differences between dream and waking reports indicated in blue. (N = 20 per group; Kruskal-Wallis test followed by two-sided Wilcoxon Rank-sum test with Bonferroni correction with α = 0.0167). (b) Rating quality measured by AUC, sensitivity and specificity, using all attributes. Notice that dream reports categorize the groups much better than waking reports. (c) Rating quality for the distinction between dream and waking reports. While reports from bipolar and control subjects can be sorted, schizophrenic subjects yield reports that fail to differentiate dream from waking.

**Figure 4:**
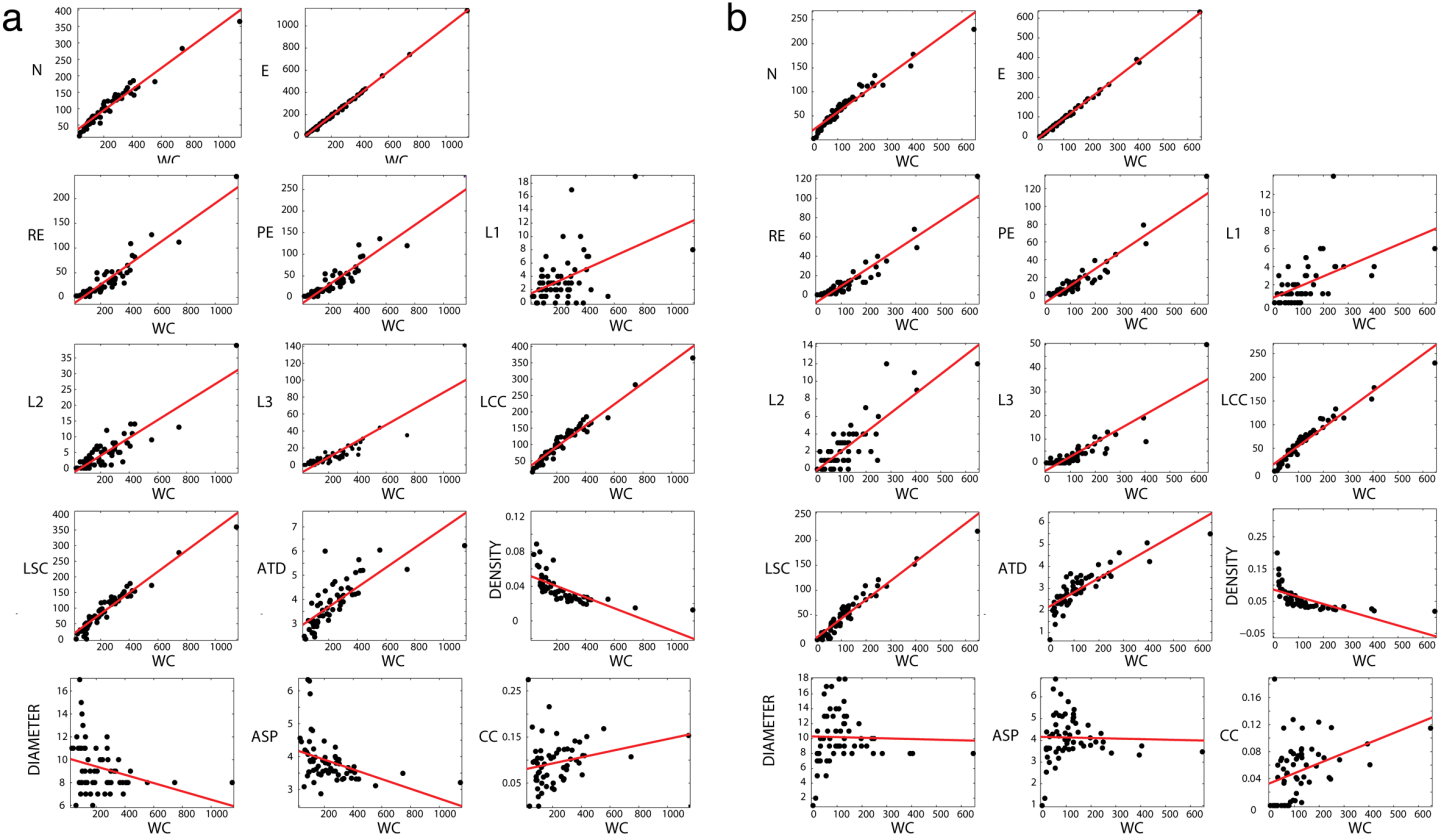
Linear correlation between SGA and word count (WC). Only L1, Density, Diameter, ASP and CC did not present a significant linear correlation with WC. (a) Dream reports. (b) Waking reports.

**Figure 5:**
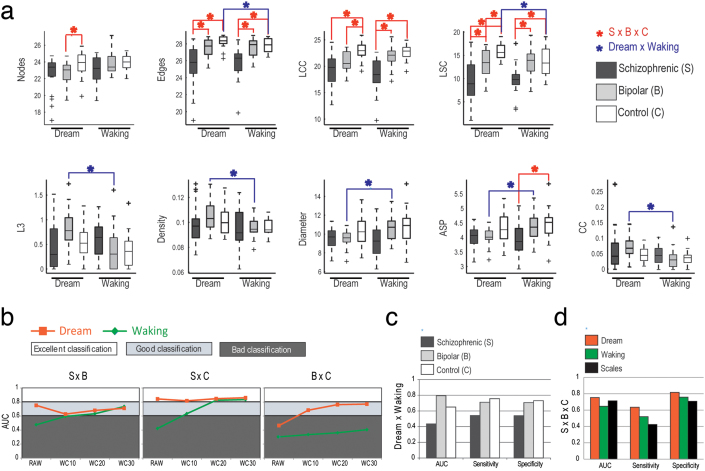
SGA controlled for verbosity differentiate psychopathological groups due to dream reports. (a) SGA boxplots for 30-word speech graphs show significant differences among schizophrenic, bipolar and control groups indicated in red, and significant differences between dream and waking reports indicated in blue (N = 20 per group for dream reports; Kruskal-Wallis test followed by two-sided Wilcoxon Rank-sum test with Bonferroni correction with α = 0.0167). Eight subjects reported on waking events using less than 30 words (for waking reports, N = 17 for the schizophrenic and control groups, and N = 18 for the bipolar group). (b) Rating quality measured by AUC, sensitivity and specificity, using all attributes. Raw data was compared with mean data obtained using analysis windows of fixed word length (10, 20 and 30 words per window). (c) The rating quality for the SGA-based distinction between dream and waking reports varies considerably across groups, reaching a maximum among bipolar subjects and a minimum among schizophrenic subjects. (d) Group sorting using dream-related SGA is better than classifications based on psychometric scores or waking-related data.

**Figure 6:**
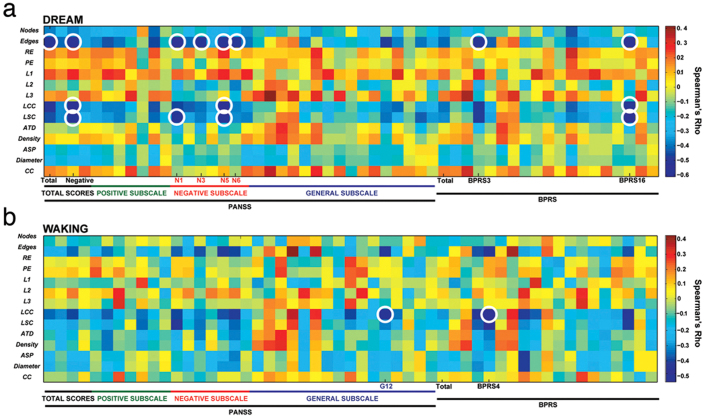
Dream-related SGA are anti-correlated with specific psychopathological symptoms. (a) Spearman's rho for correlations between individual questions of the PANSS and BPRS scales, and SGA obtained from dream reports (N = 60). Note the significant anti-correlations between SGA (E, LCC and LSC) and psychometric variables including total PANSS, PANSS negative subscale, and some negative and cognitive symptoms such as flattened affect, poor contact, difficulty in abstract thinking, loss of spontaneity or fluency in speech in PANSS; as well as emotional retraction and flattened affect in BPRS. A 30-word moving window was used for data analysis. Circles indicate P values smaller than the Bonferroni corrected α = 0.00006. (b) Same as before but for waking reports (N = 52). Note the significant anti-correlations for LCC and general psychotic symptoms measured on both scales (loss of criticism in PANSS and incoherent speech in BPRS).

**Figure 7:**
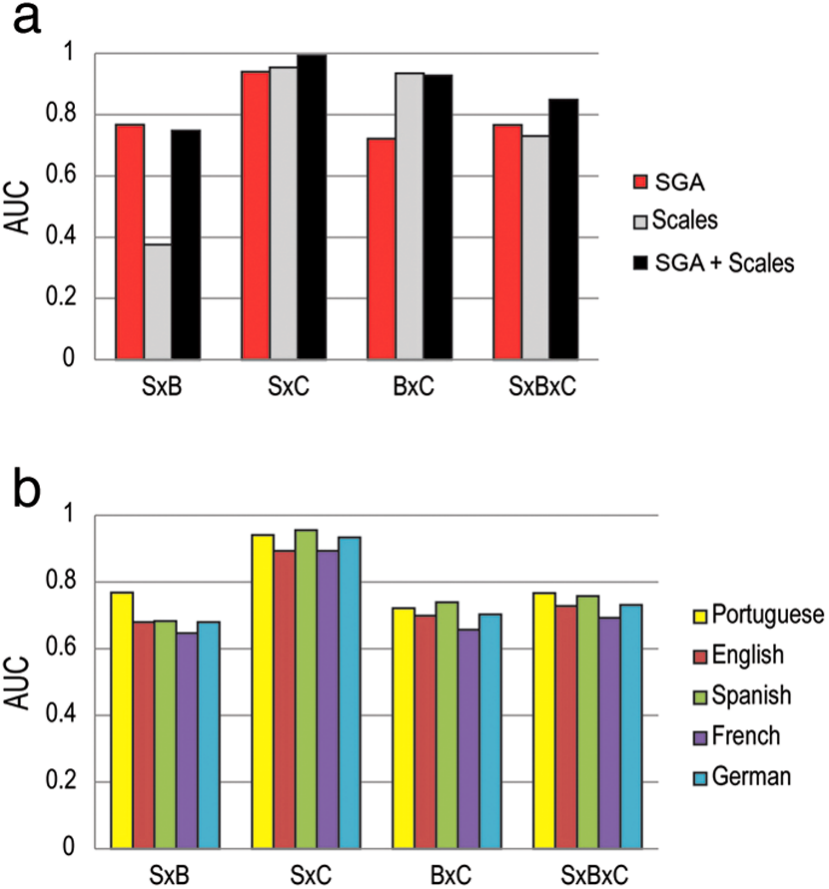
SGA improve the psychopathological sorting provided by psychometric scales. (a) Good to excellent classification of the groups was obtained using the SGA that correlated significantly with specific psychometric scores (for dream reports: E, LSC and LCC; for waking reports: LCC). Excellent classification using psychometric scales (BPRS and PANSS total scores) occurred only when sorting controls from other groups, but failed to differentiate schizophrenic from bipolar subjects. Optimal triple group classification was obtained by combining SGA and psychometric scales. Data correspond to 30-word speech graphs. (b) The SGA-based diagnosis of psychosis is invariant across the five most prevalent Western languages.

We focused our analysis on answers to two open questions: “please report a recent dream” and “please report your waking activities immediately before that dream”. Each transcribed report was represented as a word-graph^38^,^39^,^40^ in which every word was represented as a node, and the temporal link between consecutive words was represented as an edge (Figure 1a and Figure 2). To quantify graph variations, we used custom-made Java software (http://neuro.ufrn.br/softwares/speechgraphs; Supplementary Method) to calculate 14 speech graph attributes (SGA; Figure 2) comprising *general attributes*: total of nodes (N) and edges (E); *connected components*: total of nodes on the largest connected component (LCC, the maximal subgraph in which all pairs of nodes are reachable from one another in the underlying undirected subgraph), and on the largest strongly connected component (LSC, the maximal subgraph in which all pairs of nodes are reachable from one another in the directed subgraph; *recurrence attributes*: repeated edges (RE, sum of all edges linking the same pair of nodes) and parallel edges (PE, sum of all parallel edges linking the same pair of nodes given that the source node of an edge could be the target node of the parallel edge), *cycles* of one (L1, calculated as the trace of the adjacency matrix), two (L2, calculated by the trace of the squared adjacency matrix divided by two) or three (L3, calculated by the trace of the cubed adjacency matrix divided by three) nodes; *global attributes*: average total degree (ATD; given a node n, the Total Degree is the sum of “in and out” edges, and the Average Total Degree is the sum of Total Degrees of all nodes divided by the number of nodes), density *D* = 2*E*/*N*(*N* − 1), where E is the number of edges and N is the number of nodes, diameter (length of the longest shortest path between the node pairs of a network), average shortest path (ASP, average length of the shortest path between pairs of nodes of a network) and clustering coefficient (CC, given a node n, the Clustering Coefficient Map (CCMap) is the set of fractions of all n neighbors that are also neighbors of each other. Average CC is the sum of the Clustering Coefficients of all nodes in the CCMap divided by number of elements in the CCMap). The data were then analyzed in Matlab and Excel software.

### Group classification

SGAs and/or psychometric scores were used as inputs to a Naïve Bayes classifier^41^ implemented with Weka software^42^. A 10-fold cross-validation procedure was implemented to take full advantage of the sample size. Sensitivity, specificity and the area under the receiver operating characteristic curve (AUC) were used as metrics of classification quality.

### Psychometric scales

The “Positive and Negative Syndrome Scale” (PANSS)^43^ and “Brief Psychiatric Rating Scale” (BPRS)^44^ were applied during the same clinical interview from which dream and waking reports were obtained.

### Report translation

Dream and waking reports in Portuguese were translated to English, German, French, and Spanish using Google Translate.

## Author Contributions

S.R., M.C. and N.M. designed the study; N.M. collected the data; N.M., S.R., M.C., R.F. and P.P.C.M. analyzed data; R.F. and P.P.C.M. coded analysis software; N.M., S.R. and M.C. prepared figures; S.R., M.C. and N.M. wrote the manuscript.

## Supplementary Material

Supplementary Information
