# Supplementary material for: Graph analysis of dream reports is especially informative about psychosis
Source: Sci Rep. 2014 Jan 15;4:3691. doi: 10.1038/srep03691 (PMC3892182; doi:10.1038/srep03691)
Supplement: Supplementary Information [file srep03691-s1.pdf]

Supplementary Information

Supplementary Figures

Fig. S1 Page 2

Fig. S2 Page 3

Supplementary Tables

Table S1 Page 4

Table S2 Page 5

Table S3 Page 6

Table S4 Page 7

Table S5 Page 7

Supplementary Method Page 8

Supplementary Figures

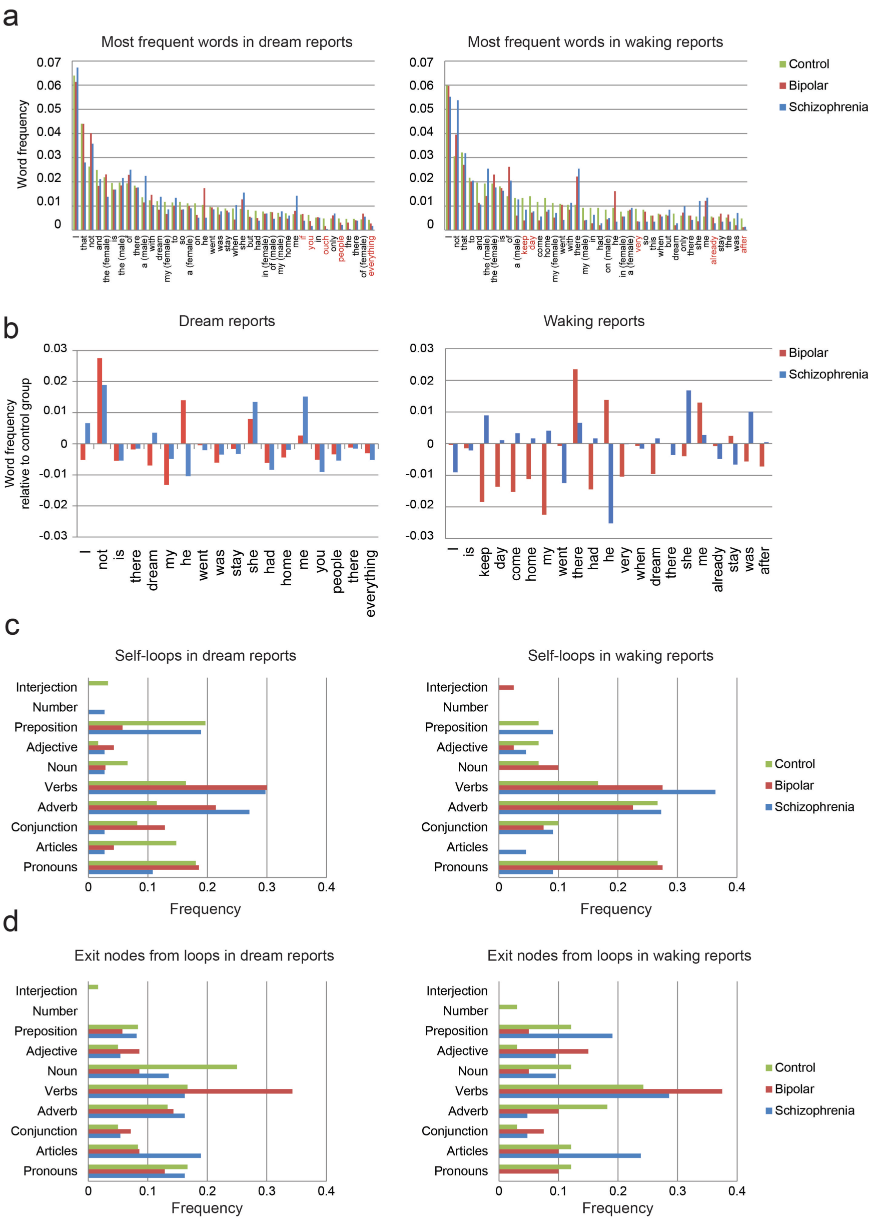

Figure S1: Semantic and grammatical properties of dream and waking reports produced by schizophrenic, bipolar and control subjects. (a) Word frequency (ratio of specific word occurrence over total word count) for the 40 most frequent words in dream and waking reports, which account for approximately 50% of the 19,625 words recorded in total. Red indicates words that are exclusive of either waking or dream reports (within the 40 most frequent words). Note that word repertoires between dream and waking reports overlap by 87.5%. (b) Relative word frequency of bipolar and schizophrenic subjects for the 40 most frequent words, excluding articles, conjunctions, prepositions, numbers and interjections. Control values were subtracted from schizophrenic and bipolar values. (c) Grammatical classification of self-loops; verbs are more prevalent in psychotic than in control subjects. (d) Grammatical classification of words that follow self-loops (exit words); verbs are more prevalent in bipolar than in control subjects.

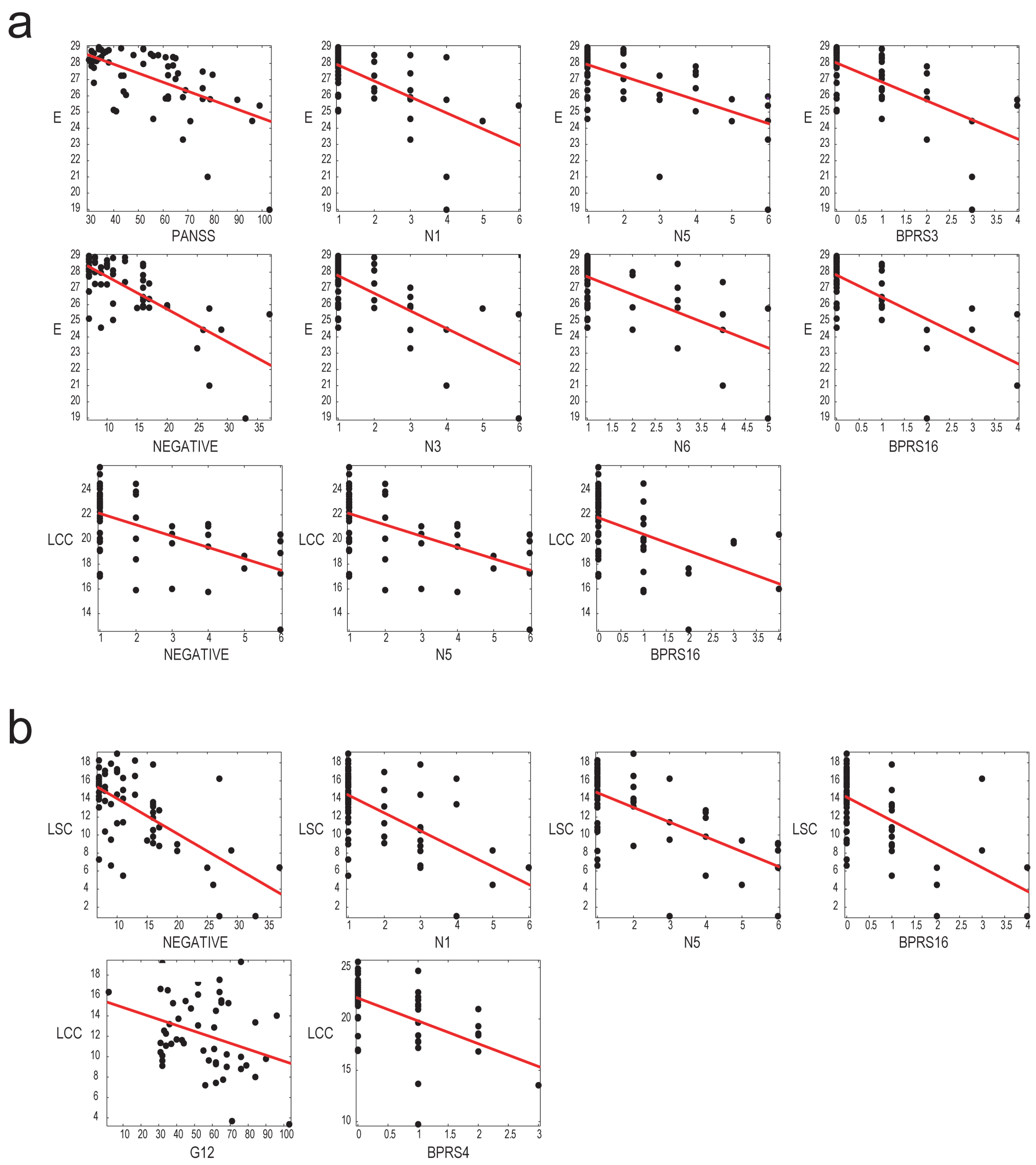

Figure S2: Linear correlation of SGA and individual questions of the psychometric scales. Plots correspond to the significant Spearman's correlations in Figure 3. **(a)** Dream reports. **(b)** Waking reports.

Supplementary Tables

		Psychotic Subjects		Controls
**Demographic Characteristics**		**Schizophrenia**	**Bipolar Type I**	
**Age (Years)**		34.10 ± 9.60	38.15 ± 12.44	35.05 ± 10.93
**Sex**	Male	80%	70%	45%
	Female	20%	30%	55%
**Years of Education**		5.50 ± 2.85	7.30 ± 3.15	6.90 ± 3.11
**Marital Status**	Married	20%	45%	65%
	Previously Married	25%	20%	10%
	Never Married	55%	35%	25%
**Psychiatric Assesment**				
**Scales**	PANSS	69 ± 16.59	61.10 ± 15.49	36.15 ± 6.43
	BPRS	16.40 ± 7.26	15.60 ± 7.83	3.95 ± 3.72
**Medication**	Typical Antipsychotic	85%	55%	0
	Atypical Antipsychotic	25%	20%	0
	Mood Stabilizer	15%	55%	0
	Benzodiazepine	25%	25%	15%
	Antidepressants	0%	20%	20%
**Age of Onset**		22.5 ± 7.67	27.1 ± 9.49	36.8 ± 7.96
**Disease Duration**		12 ± 8.3	11.6 ± 9.94	1.24 ± 1.4

Table S1: Socio-demographic and psychiatric information about the groups investigated. Age (years), years of education, total score of PANSS and BPRS and frequency of sex, marital status and medication for the groups studied. Mean and standard deviation are indicated. All subjects were Brazilian. Control subjects were non-psychotic individuals with depression (N=5), generalized anxiety disorder (N=2), one past episode of post-traumatic stress disorder (N=1), various symptoms of mood/anxiety disorder without reaching diagnostic criteria (N=11), plus one healthy individual.

Initials	Group	Content	WC	N	E	RE	PE	L1	L2	L3	LCC	LSC	ATD	Density	Diameter	ASP	CC	PANSS	BPRS
AN	schizophrenia	DREAM	30	17	21	3	3	1	0	0	16	1	2.4706	0.1250	11	4.5583	0.2745	78	18
CF	schizophrenia	DREAM	243	92	218	52	62	10	12	10	89	71	4.7391	0.0349	10	3.6412	0.1230	62	13
CLG	schizophrenia	DREAM	91	57	85	6	7	1	1	1	57	48	2.9825	0.0482	7	3.5345	0.0561	66	26
CRIS	schizophrenia	DREAM	81	38	73	12	14	3	2	3	37	33	3.8421	0.0797	8	3.4144	0.1189	76	22
DB	schizophrenia	DREAM	95	62	89	7	9	2	2	2	57	47	2.8710	0.0412	11	4.4586	0.0377	62	13
EL	schizophrenia	DREAM	93	52	81	7	7	4	1	5	51	36	3.1154	0.0528	8	3.5318	0.0930	96	29
FA	schizophrenia	DREAM	198	113	177	11	17	2	7	2	113	95	3.1327	0.0250	11	4.4722	0.0391	68	16
FF	schizophrenia	DREAM	123	77	117	2	3	1	1	2	75	67	3.0390	0.0386	8	3.5827	0.0277	58	12
FJJ	schizophrenia	DREAM	100	65	84	8	10	4	3	1	61	18	2.5846	0.0337	14	5.9087	0.0205	56	11
FV	schizophrenia	DREAM	102	54	66	8	8	3	0	0	38	1	2.4444	0.0384	9	3.8506	0.0000	103	30
IT	schizophrenia	DREAM	117	73	105	10	13	1	3	4	66	45	2.8767	0.0346	11	4.7921	0.1204	44	5
JA	schizophrenia	DREAM	384	179	377	50	58	0	8	25	176	170	4.2123	0.0200	8	3.5674	0.0936	55	15
JC	schizophrenia	DREAM	107	64	95	13	15	0	2	2	64	35	2.9688	0.0397	13	4.8309	0.0741	41	4
JFA	schizophrenia	DREAM	84	55	72	8	8	3	0	1	53	9	2.6182	0.0411	17	6.3389	0.0563	71	17
JM	schizophrenia	DREAM	64	40	55	5	5	0	0	1	40	26	2.7500	0.0641	12	4.1987	0.0955	62	9
JPS	schizophrenia	DREAM	39	28	33	3	3	1	0	0	27	18	2.3571	0.0767	11	4.4530	0.0000	99	24
LL	schizophrenia	DREAM	58	32	50	4	6	0	2	3	31	30	3.1250	0.0887	6	3.0860	0.1716	90	25
MG	schizophrenia	DREAM	206	94	183	25	30	4	5	10	94	81	3.8936	0.0341	9	3.8115	0.1065	61	11
ML	schizophrenia	DREAM	281	143	269	21	25	3	5	17	143	132	3.7622	0.0237	10	3.7992	0.1231	64	14
PS	schizophrenia	DREAM	85	53	69	7	5	3	0	3	47	25	2.6038	0.0443	11	4.0685	0.0639	68	14
AB	bipolar	DREAM	335	146	327	50	55	0	5	23	146	137	4.4795	0.0257	9	3.3350	0.1215	56	12
DP	bipolar	DREAM	136	77	128	12	14	4	3	4	77	70	3.3247	0.0376	8	3.9798	0.0631	80	22
EMN	bipolar	DREAM	410	141	398	109	122	7	14	19	139	139	5.6454	0.0273	8	3.5170	0.1100	61	17
FC	bipolar	DREAM	142	73	140	13	18	3	5	8	73	68	3.8356	0.0453	10	3.6157	0.0913	35	4
FM	bipolar	DREAM	301	141	275	36	42	3	7	13	141	124	3.9007	0.0233	9	3.9110	0.0880	76	24
IS	bipolar	DREAM	303	131	273	39	36	17	8	16	130	114	4.1679	0.0258	8	3.7821	0.1241	69	17
JB	bipolar	DREAM	325	131	318	52	56	5	5	12	129	123	4.8550	0.0302	9	3.3096	0.1020	48	5
JF	bipolar	DREAM	95	63	82	2	2	5	0	4	63	29	2.6032	0.0384	15	6.2954	0.1042	40	6
JG	bipolar	DREAM	276	136	244	31	33	6	5	7	136	112	3.5882	0.0223	11	4.2880	0.0566	45	8
JJDM	bipolar	DREAM	124	75	115	6	5	7	1	3	74	57	3.0667	0.0371	12	4.2325	0.0497	43	8
JMR	bipolar	DREAM	365	164	344	29	38	10	11	19	164	153	4.1951	0.0221	8	3.6652	0.0998	62	18
JMS	bipolar	DREAM	355	156	343	43	45	3	2	22	156	146	4.3974	0.0244	10	3.4878	0.1413	84	32
LA	bipolar	DREAM	176	74	161	37	41	3	6	15	74	67	4.3514	0.0433	8	3.4447	0.2158	65	12
MCAAG	bipolar	DREAM	1147	365	1137	245	280	8	39	141	365	360	6.2301	0.0128	8	3.2098	0.1534	38	6
MDRN	bipolar	DREAM	287	126	280	40	48	2	8	11	126	117	4.4444	0.0292	8	3.2863	0.0847	65	17
MHDA	bipolar	DREAM	279	133	271	52	56	1	4	10	132	129	4.0752	0.0244	8	3.9619	0.0595	64	18
MLS	bipolar	DREAM	228	96	222	46	51	4	6	14	96	88	4.6250	0.0366	10	3.5011	0.1578	84	28
OF	bipolar	DREAM	126	58	121	17	19	2	2	7	58	54	4.1724	0.0605	7	3.2099	0.1635	52	13
SBC	bipolar	DREAM	75	44	68	5	7	2	2	5	44	30	3.0909	0.0624	11	4.1438	0.0973	76	23
WEI	bipolar	DREAM	188	97	165	17	20	2	3	9	91	72	3.4021	0.0307	9	3.8833	0.1035	79	22
AA	control	DREAM	275	134	266	36	37	0	1	9	134	123	3.9701	0.0257	12	4.0009	0.0570	33	3
ARS	control	DREAM	388	147	376	65	71	8	8	25	146	138	5.1156	0.0277	7	3.3427	0.1390	35	4
CESQ	control	DREAM	122	63	118	15	19	1	4	8	63	51	3.7460	0.0502	11	3.9304	0.0843	38	5
DLSN	control	DREAM	175	56	168	50	55	5	7	7	56	54	6.0000	0.0701	6	2.8708	0.1098	31	1
DPC	control	DREAM	228	123	220	21	21	2	1	14	123	119	3.5772	0.0263	10	3.7860	0.1151	37	3
ERS	control	DREAM	260	122	255	30	35	3	5	9	121	120	4.1803	0.0294	7	3.5623	0.0644	32	1
FM	control	DREAM	438	166	432	82	96	2	14	31	166	154	5.2048	0.0244	9	3.3177	0.1509	36	4
FRS	control	DREAM	210	103	204	21	23	3	2	4	103	98	3.9612	0.0339	7	3.5839	0.0363	32	1
GQA	control	DREAM	421	161	418	85	94	7	11	27	161	156	5.1925	0.0246	8	3.3184	0.1095	52	11
JO	control	DREAM	160	88	146	13	18	3	6	4	82	75	3.3182	0.0327	7	3.4718	0.0673	32	2
JS	control	DREAM	556	182	550	127	136	1	9	44	182	173	6.0440	0.0251	8	3.1152	0.1682	31	1
LLS	control	DREAM	276	136	274	30	35	2	5	9	136	136	4.0294	0.0258	8	3.5568	0.0836	52	14
LVM	control	DREAM	121	76	118	5	5	1	0	6	76	73	3.1053	0.0393	9	3.8533	0.1037	43	9
MFNCL	control	DREAM	746	283	742	112	120	19	13	35	283	278	5.2438	0.0151	8	3.4907	0.1072	34	3
MGPCS	control	DREAM	100	65	94	5	5	1	0	3	60	52	2.8923	0.0423	8	3.8842	0.0722	31	1
ML	control	DREAM	189	99	180	17	19	0	2	10	99	91	3.6364	0.0332	8	3.5521	0.1051	32	2
MSB	control	DREAM	204	121	203	15	16	1	1	6	121	117	3.3554	0.0256	9	3.9751	0.0500	34	3
OBR	control	DREAM	406	185	394	55	62	5	7	12	185	179	4.2595	0.0192	8	3.6938	0.0680	30	0
OS	control	DREAM	157	80	142	14	14	2	1	3	78	70	3.5500	0.0399	9	3.7060	0.0426	44	9
RFS	control	DREAM	297	139	287	28	34	4	6	9	139	133	4.1295	0.0260	7	3.4429	0.0792	34	2

Table S2: Individual SGA and psychometric data for dream reports (N=60).

Initials	Group	Content	WC	N	E	RE	PE	L1	L2	L3	LCC	LSC	ATD	Density	Diameter	ASP	CC
AN	schizophrenia	WAKE	3	3	1	0	0	0	0	0	2	1	0.6667	0.3333	1	1.0000	0.0000
CF	schizophrenia	WAKE	138	78	117	15	20	4	5	5	77	43	3.0000	0.0310	13	4.7792	0.0424
CLG	schizophrenia	WAKE	49	38	45	1	2	1	1	1	27	9	2.3684	0.0597	13	5.1510	0.0702
CRIS	schizophrenia	WAKE	86	43	79	13	16	2	3	3	43	34	3.6744	0.0676	8	3.5305	0.0800
DB	schizophrenia	WAKE	129	76	112	6	7	5	3	6	67	52	2.9474	0.0351	9	3.9118	0.0842
EL	schizophrenia	WAKE	249	134	238	21	26	4	6	13	133	121	3.5522	0.0233	10	3.9183	0.1155
FA	schizophrenia	WAKE	129	73	118	11	13	1	2	4	68	57	3.2329	0.0396	10	3.9083	0.0758
FF	schizophrenia	WAKE	127	79	111	12	13	1	1	2	77	63	2.8101	0.0315	12	4.8869	0.0328
FJJ	schizophrenia	WAKE	27	22	21	1	1	0	0	0	19	8	1.9091	0.0866	8	3.6374	0.0000
FV	schizophrenia	WAKE	56	40	35	1	1	2	1	1	13	4	1.7500	0.0410	5	2.7179	0.0542
IT	schizophrenia	WAKE	219	112	199	18	20	1	2	10	109	89	3.5536	0.0286	9	3.9201	0.0708
JA	schizophrenia	WAKE	170	85	153	19	19	3	2	7	83	69	3.6000	0.0367	9	3.7346	0.0835
JC	schizophrenia	WAKE	95	48	79	12	15	1	3	2	37	29	3.2917	0.0559	11	3.6486	0.1274
JFA	schizophrenia	WAKE	48	34	39	4	6	0	2	0	27	4	2.2941	0.0588	16	6.3533	0.0000
JM	schizophrenia	WAKE	84	57	70	4	5	0	1	1	55	30	2.4561	0.0407	13	5.0949	0.0053
JPS	schizophrenia	WAKE	20	14	14	1	1	3	1	0	8	2	2.0000	0.1099	5	2.5357	0.0000
LL	schizophrenia	WAKE	72	49	55	4	5	0	1	0	44	15	2.2449	0.0425	14	5.1342	0.0000
MG	schizophrenia	WAKE	107	69	99	3	6	1	3	3	69	56	2.8696	0.0392	14	4.5780	0.0415
ML	schizophrenia	WAKE	111	62	108	10	11	1	1	1	62	62	3.4839	0.0508	9	3.5764	0.0393
PS	schizophrenia	WAKE	33	20	26	2	2	2	0	0	17	10	2.6000	0.1158	9	3.6324	0.0417
AB	bipolar	WAKE	129	79	117	14	15	3	1	1	79	49	2.9620	0.0321	13	5.1951	0.0045
DP	bipolar	WAKE	20	17	18	0	0	0	0	0	17	5	2.1176	0.1324	8	3.5735	0.0000
EMN	bipolar	WAKE	96	58	90	13	13	2	0	2	57	52	3.1034	0.0454	11	4.3296	0.0606
FC	bipolar	WAKE	24	22	15	0	0	0	0	0	10	1	1.3636	0.0649	7	3.2222	0.0000
FM	bipolar	WAKE	240	118	218	29	28	14	4	4	118	89	3.6949	0.0255	10	4.3139	0.0415
IS	bipolar	WAKE	108	74	102	4	8	0	4	1	74	58	2.7568	0.0348	18	5.7734	0.0180
JB	bipolar	WAKE	198	94	192	34	39	6	7	11	94	81	4.0851	0.0336	9	3.7694	0.1236
JF	bipolar	WAKE	132	81	121	10	14	3	4	7	77	53	2.9877	0.0321	18	5.4111	0.1149
JG	bipolar	WAKE	62	45	55	2	2	1	0	0	43	36	2.4444	0.0525	11	4.3909	0.0000
JJDM	bipolar	WAKE	71	47	65	5	6	1	1	1	46	36	2.7660	0.0537	10	4.1942	0.0482
JMR	bipolar	WAKE	33	25	29	1	1	2	0	0	23	15	2.3200	0.0867	10	4.1660	0.0600
JMS	bipolar	WAKE	59	46	56	1	1	1	0	0	46	30	2.4348	0.0522	13	5.2531	0.0000
LA	bipolar	WAKE	103	70	97	3	6	0	3	1	69	63	2.7714	0.0377	9	3.8602	0.0073
MCAAG	bipolar	WAKE	648	230	631	123	134	6	12	50	230	218	5.4870	0.0186	8	3.4691	0.1147
MDRN	bipolar	WAKE	59	37	57	6	7	0	1	1	37	34	3.0811	0.0751	7	3.4595	0.0360
MHDA	bipolar	WAKE	197	112	192	13	17	1	4	8	112	109	3.4286	0.0280	10	4.1100	0.0757
MLS	bipolar	WAKE	148	83	144	24	28	1	4	4	83	72	3.4699	0.0338	11	4.3682	0.0602
OF	bipolar	WAKE	114	63	103	12	15	1	3	2	63	52	3.2698	0.0445	11	4.2412	0.0738
SBC	bipolar	WAKE	69	38	67	9	13	1	4	1	37	36	3.5263	0.0754	7	3.1742	0.0666
WEI	bipolar	WAKE	128	74	109	12	11	3	0	1	73	45	2.9459	0.0352	15	5.0190	0.0824
AA	control	WAKE	188	115	178	13	14	6	4	5	113	93	3.0957	0.0241	12	4.6914	0.0586
ARS	control	WAKE	160	89	157	18	22	2	4	4	88	85	3.5281	0.0340	8	3.8670	0.0347
CESQ	control	WAKE	21	19	17	0	0	0	0	0	17	4	1.7895	0.0994	11	4.3824	0.0000
DLSN	control	WAKE	58	41	54	5	5	4	2	0	38	28	2.6341	0.0549	13	4.7141	0.0332
DPC	control	WAKE	95	59	83	5	7	0	2	0	59	46	2.8136	0.0444	9	4.0333	0.0078
ERS	control	WAKE	96	62	90	8	10	1	2	2	62	34	2.9032	0.0418	14	5.2073	0.0769
FM	control	WAKE	282	114	264	35	46	4	12	12	114	108	4.6316	0.0332	8	3.4102	0.0678
FRS	control	WAKE	57	45	54	4	4	1	0	3	45	20	2.4000	0.0495	17	6.8111	0.1148
GQA	control	WAKE	394	154	391	68	79	3	11	19	154	152	5.0779	0.0262	8	3.3231	0.0914
JO	control	WAKE	69	45	66	5	5	0	0	1	45	42	2.9333	0.0616	9	3.6253	0.0459
JS	control	WAKE	246	113	237	40	38	4	1	6	113	109	4.1947	0.0308	8	3.6487	0.0392
LLS	control	WAKE	127	69	124	13	15	3	3	3	69	68	3.5942	0.0452	8	3.6138	0.0760
LVM	control	WAKE	108	72	106	3	4	1	1	4	72	67	2.9444	0.0395	9	4.0391	0.0690
MFNCL	control	WAKE	130	74	123	14	16	5	3	6	74	69	3.3243	0.0378	12	5.0574	0.0709
MGPCS	control	WAKE	45	31	41	3	4	0	1	1	31	11	2.6452	0.0796	10	4.2796	0.0667
ML	control	WAKE	78	61	71	1	2	2	1	0	55	28	2.3279	0.0366	17	6.1226	0.0000
MSB	control	WAKE	404	178	376	49	58	4	9	9	178	163	4.2247	0.0199	8	3.7397	0.0605
OBR	control	WAKE	20	16	19	1	1	0	0	2	16	15	2.3750	0.1500	7	3.6417	0.1875
OS	control	WAKE	14	6	6	0	2	1	2	0	3	3	2.0000	0.2000	2	1.3333	0.0000
RFS	control	WAKE	32	22	29	1	2	1	1	0	19	17	2.6364	0.1126	7	2.9825	0.0000

Table S3: Individual SGA and psychometric data for waking reports (N=60).

DREAM	KW	SxB	SxC	BxC	WC 10	KW	SxB	SxC	BxC	WC 20	KW	SxB	SxC	BxC	WC 30	KW	SxB	SxC	BxC
Nodes	0.0003	0.0013	0.0003	0.5427	Nodes	0.0096	0.6750	0.0066	0.0133	Nodes	0.0198	0.9676	0.0200	0.0133	Nodes	0.0231	0.6168	0.0385	0.0106
Edges	0.0001	0.0004	0.0001	0.6359	Edges	0.0000	0.0015	0.0000	0.0315	Edges	0.0000	0.0015	0.0000	0.0275	Edges	0.0000	0.0013	0.0000	0.0275
RE	0.0006	0.0021	0.0004	1	RE	0.2603	0.5608	0.4734	0.0764	RE	0.0683	0.2085	0.4407	0.0167	RE	0.0557	0.1199	0.5609	0.0167
PE	0.0010	0.0029	0.0007	0.9568	PE	0.4077	0.3648	0.6554	0.2085	PE	0.1267	0.1136	0.6359	0.0679	PE	0.1106	0.1199	0.7764	0.0468
L1	0.0388	0.0133	0.4097	0.0985	L1	0.2115	0.4484	0.4896	0.0601	L1	0.1966	0.3937	0.4400	0.0638	L1	0.1197	0.6356	0.1888	0.0385
L2	0.0250	0.0078	0.0560	0.6637	L2	0.4301	0.3021	0.9673	0.2389	L2	0.3446	0.2429	0.7961	0.1895	L2	0.3811	0.2540	0.7544	0.2286
L3	0.0001	0.0001	0.0003	0.6160	L3	0.0625	0.0292	0.3929	0.1017	L3	0.0381	0.0214	0.2785	0.0720	L3	0.0227	0.0192	0.3430	0.0256
LCC	0.0002	0.0010	0.0002	0.5607	LCC	0.0000	0.0155	0.0000	0.0056	LCC	0.0000	0.0239	0.0001	0.0033	LCC	0.0002	0.0909	0.0002	0.0031
LSC	0.0001	0.0008	0.0000	0.3368	LSC	0.0539	0.0275	0.0601	0.6750	LSC	0.0001	0.0040	0.0001	0.0565	LSC	0.0000	0.0051	0.0001	0.0066
ATD	0.0001	0.0003	0.0002	0.9892	ATD	0.0036	0.0028	0.0060	0.6949	ATD	0.0186	0.0106	0.0294	0.4903	ATD	0.0591	0.0315	0.0810	0.4094
Density	0.0101	0.0133	0.0066	0.9246	Density	0.0583	0.0223	0.1264	0.3793	Density	0.1891	0.0720	0.4407	0.3104	Density	0.2723	0.1556	0.4735	0.2503
Diameter	0.0143	0.1717	0.0076	0.0571	Diameter	0.0000	0.0439	0.0000	0.0060	Diameter	0.0011	0.0601	0.0007	0.0207	Diameter	0.2295	0.6073	0.1895	0.1264
ASP	0.0179	0.0499	0.0066	0.4407	ASP	0.0000	0.0385	0.0000	0.0071	ASP	0.0006	0.0468	0.0004	0.0179	ASP	0.1900	0.7557	0.1404	0.1075
CC	0.1809	0.0858	0.4903	0.2085	CC	0.1041	0.0676	0.6551	0.0764	CC	0.1054	0.0961	0.7352	0.0499	CC	0.0606	0.0764	0.8181	0.0223
WC	0.0001	0.0005	0.0001	0.8392															
**WAKING**	**KW**	**SxB**	**SxC**	**BxC**	**WC 10**	**KW**	**SxB**	**SxC**	**BxC**	**WC 20**	**KW**	**SxB**	**SxC**	**BxC**	**WC 30**	**KW**	**SxB**	**SxC**	**BxC**
Nodes	0.6393	0.3647	0.5338	0.7867	Nodes	0.1550	0.0840	0.1124	1	Nodes	0.1657	0.2551	0.0577	0.5273	Nodes	0.2575	0.3469	0.1480	0.3062
Edges	0.6397	0.4093	0.4407	0.9246	Edges	0.0004	0.0033	0.0002	0.4171	Edges	0.0001	0.0016	0.0000	0.4737	Edges	0.0002	0.0020	0.0001	0.4577
RE	0.7897	0.4975	0.6541	0.9136	RE	0.2252	0.4646	0.0936	0.3150	RE	0.4412	0.4822	0.2251	0.5177	RE	0.3986	0.9211	0.3261	0.1866
PE	0.7801	0.4723	0.7042	0.8284	PE	0.4333	0.2855	0.2731	0.7149	PE	0.3839	0.3836	0.1938	0.5551	PE	0.3978	0.7917	0.3432	0.1757
L1	0.7878	0.8559	0.4881	0.6879	L1	0.9791	0.9437	0.9887	0.8062	L1	0.9719	0.8764	0.9766	0.8207	L1	0.6658	0.4976	0.3974	0.8686
L2	0.9157	0.7933	0.6986	0.8794	L2	0.2304	0.0926	0.3901	0.3953	L2	0.1305	0.0494	0.2420	0.4019	L2	0.2288	0.0915	0.4484	0.3621
L3	0.8400	0.9339	0.6599	0.5915	L3	0.5735	0.3140	0.6803	0.5137	L3	0.4448	0.2096	0.6479	0.4585	L3	0.1746	0.0969	0.1322	0.8028
LCC	0.3811	0.1985	0.2912	0.8498	LCC	0.0016	0.0062	0.0009	0.4569	LCC	0.0002	0.0014	0.0001	0.4480	LCC	0.0001	0.0012	0.0001	0.2038
LSC	0.2662	0.1633	0.1676	0.8924	LSC	0.5051	0.2436	0.7254	0.4818	LSC	0.0295	0.0416	0.0131	0.7466	LSC	0.0106	0.0116	0.0084	0.7791
ATD	0.5415	0.3793	0.3169	0.8817	ATD	0.1213	0.0946	0.0657	0.8392	ATD	0.0839	0.0699	0.0471	0.7895	ATD	0.3653	0.2548	0.2025	0.9342
Density	0.7659	0.4903	0.7764	0.6554	Density	0.3571	0.1914	0.2551	0.7764	Density	0.3169	0.1820	0.2093	1	Density	0.8474	0.6322	0.6543	0.8301
Diameter	0.6210	0.6729	0.5481	0.3610	Diameter	0.0052	0.0204	0.0021	0.3942	Diameter	0.0013	0.0054	0.0013	0.1686	Diameter	0.0221	0.0200	0.0175	0.6679
ASP	0.7031	0.4249	0.8604	0.5609	ASP	0.0040	0.0140	0.0019	0.4094	ASP	0.0007	0.0043	0.0006	0.1914	ASP	0.0207	0.0200	0.0159	0.6322
CC	0.9604	1	0.8383	0.7964	CC	0.6945	0.4205	0.6708	0.6384	CC	0.6039	0.4112	0.9415	0.3817	CC	0.3343	0.1917	0.2553	0.6555
WC	0.7588	0.4902	0.6456	0.7557															

Table S4: P values of non-parametrical statistical analysis comparing SGA for raw data (full reports) and fixed WC data (graphs of 10, 20 and 30 words). P values using Kruskal-Wallis test on SxBxC (differences among groups tested together), considering $P < 0.05$ and Wilcoxon Ranksum test with Bonferroni correction (for 3 pairwise comparisons, α=0.0167). Red indicates statistically significant differences.

DREAM															
S x B x C	AUC	Sensitivity	Specificity	S x B	AUC	Sensitivity	Specificity	S x C	AUC	Sensitivity	Specificity	B x C	AUC	Sensitivity	Specificity
RAW	0.645	0.4	0.7	RAW	0.752	0.7	0.7	RAW	0.843	0.7	0.7	RAW	0.464	0.475	0.475
WC 10	0.674	0.5	0.75	WC 10	0.628	0.6	0.6	WC 10	0.817	0.8	0.8	WC 10	0.683	0.65	0.65
WC 20	0.71	0.517	0.758	WC 20	0.68	0.675	0.675	WC 20	0.847	0.775	0.775	WC 20	0.763	0.625	0.625
WC 30	0.731	0.55	0.775	WC 30	0.71	0.65	0.65	WC 30	0.86	0.775	0.793775	WC 30	0.77	0.65	0.65
**WAKING**															
**S x B x C**	**AUC**	**Sensitivity**	**Specificity**	**S x B**	**AUC**	**Sensitivity**	**Specificity**	**S x C**	**AUC**	**Sensitivity**	**Specificity**	**B x C**	**AUC**	**Sensitivity**	**Specificity**
RAW	0.419	0.267	0.633	RAW	0.475	0.55	0.55	RAW	0.423	0.575	0.575	RAW	0.303	0.375	0.375
WC 10	0.488	0.407	0.699	WC 10	0.595	0.615	0.603	WC 10	0.634	0.641	0.638	WC 10	0.335	0.425	0.425
WC 20	0.601	0.414	0.704	WC 20	0.632	0.615	0.608	WC 20	0.825	0.763	0.763	WC 20	0.361	0.41	0.419
WC 30	0.632	0.5	0.749	WC 30	0.735	0.657	0.657	WC 30	0.834	0.794	0.794	WC 30	0.405	0.514	0.512

Table S5: Classification quality measured by AUC, Sensitivity and Specificity. A Naïve Bayes classifier was used to split the 3 groups (SxBxC), or separately sort SxB, SxC, and BxC, using all SGA as inputs.

Supplementary Method

Customized software for the graph analysis of text (*SpeechGraphs*)

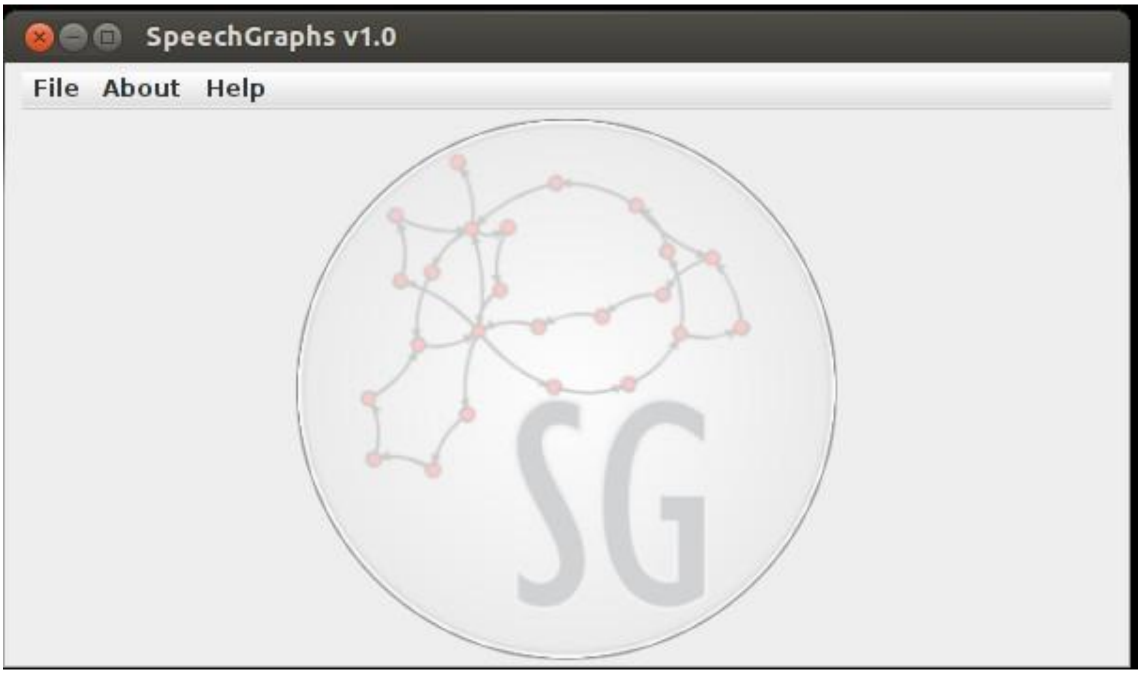

Speechgraphs is a graph-theoretical analysis tool that uses text as input and graph features as output. This customized software plots graphs and calculates graph attributes with moving windows of fixed word length. The *Speechgraphs* software was developed at the Brain Institute of the Federal University of Rio Grande do Norte (Natal, Brazil), by R. Furtado, P.P.C. Maia, N.B. Mota, S. Ribeiro, M. Copelli, and D.F. Slezak. The software and a complete user's guide can be directly downloaded from the website: http://neuro.ufrn.br/research/softwares/speechgraphs.
